# RRx-001: a chimeric triple action NLRP3 inhibitor, Nrf2 inducer, and nitric oxide superagonist

**DOI:** 10.3389/fonc.2023.1204143

**Published:** 2023-05-29

**Authors:** Bryan Oronsky, Lori Takahashi, Richard Gordon, Pedro Cabrales, Scott Caroen, Tony Reid

**Affiliations:** ^1^ Drug Development, EpicentRx, Torrey Pines, CA, United States; ^2^ Department of Translational Neuroscience, University of Queensland Centre for Clinical Research, Brisbane, QLD, Australia; ^3^ Department of Bioengineering, University of California at San Diego, La Jolla, CA, United States

**Keywords:** RRx-001, NLRP3 inflammasome, Nrf2, KEAP1, nitric oxide, antibody drug conjugate (ADC), NFkB

## Abstract

RRx-001 is a shape shifting small molecule with Fast Track designation for the prevention/amelioration of chemoradiation-induced severe oral mucositis (SOM) in newly diagnosed Head and Neck cancer. It has been intentionally developed or “engineered” as a chimeric single molecular entity that targets multiple redox-based mechanisms. Like an antibody drug conjugate (ADC), RRx-001 contains, at one end a “targeting” moiety, which binds to the NLRP3 inflammasome and inhibits it as well as Kelch-like ECH-associated protein 1 (KEAP1), the negative regulator of Nrf2, and, at the other end, a conformationally constrained, dinitro containing 4 membered ring, which fragments under conditions of hypoxia and reduction to release therapeutically active metabolites i.e., the payload. This “payload”, which is delivered specifically to hypoperfused and inflamed areas, includes nitric oxide, nitric oxide related species and carbon-centered radicals. As observed with ADCs, RRx-001 contains a backbone amide “linker” attached to a binding site, which correlates with the F_ab_ region of an antibody, and to the dinitroazetidine payload, which is microenvironmentally activated. However, unlike ADCs, whose large size impacts their pharmacokinetic properties, RRx-001 is a nonpolar small molecule that easily crosses cell membranes and the blood brain barrier (BBB) and distributes systemically. This short review is organized around the *de novo* design and *in vivo* pro-oxidant/pro-inflammatory and antioxidant/anti-inflammatory activity of RRx-001, which, in turn, depends on the reduced to oxidized glutathione ratio and the oxygenation status of tissues.

## 1 Introduction

This short review covers the *de novo* design of the chimeric or hybrid therapy, RRx-001, also referred to by its chemical acronym, ABDNAZ, for alpha bromodinitroazetidine, and its United States Adopted Names (USAN) generic, non-proprietary, first-in-class name, nibrozetone.

Increasingly, it has become clear that many disorders especially progressive ones like several types of cancer, neurodegenerative diseases, congestive heart failure, diabetes, and kidney disease are too complex and too multifactorial to be successfully treated by a single medication or therapy (1). This provides support for polypharmacology of which perhaps the most successful example is the triple therapy cocktail for human immunodeficiency virus (HIV). However, combination therapies are potentially subject to drug-drug interactions, additive toxicities, and the development of resistance, which limit their usefulness.

An alternative to combination of two or more therapies administered separately is multi-targeted drugs particularly those obtained through conjugation of two or more pharmacophores having specific pharmacological activities and rendered potentially more effective and less toxic than in isolation due to the avoidance of different bioavailabilities, pharmacokinetics, metabolism, and drug-drug interactions (2). Several small molecule chimeric drugs are in development; however, to best knowledge, one of the most clinically advanced is RRx-001 on which this review focuses.

**Figure 1:**
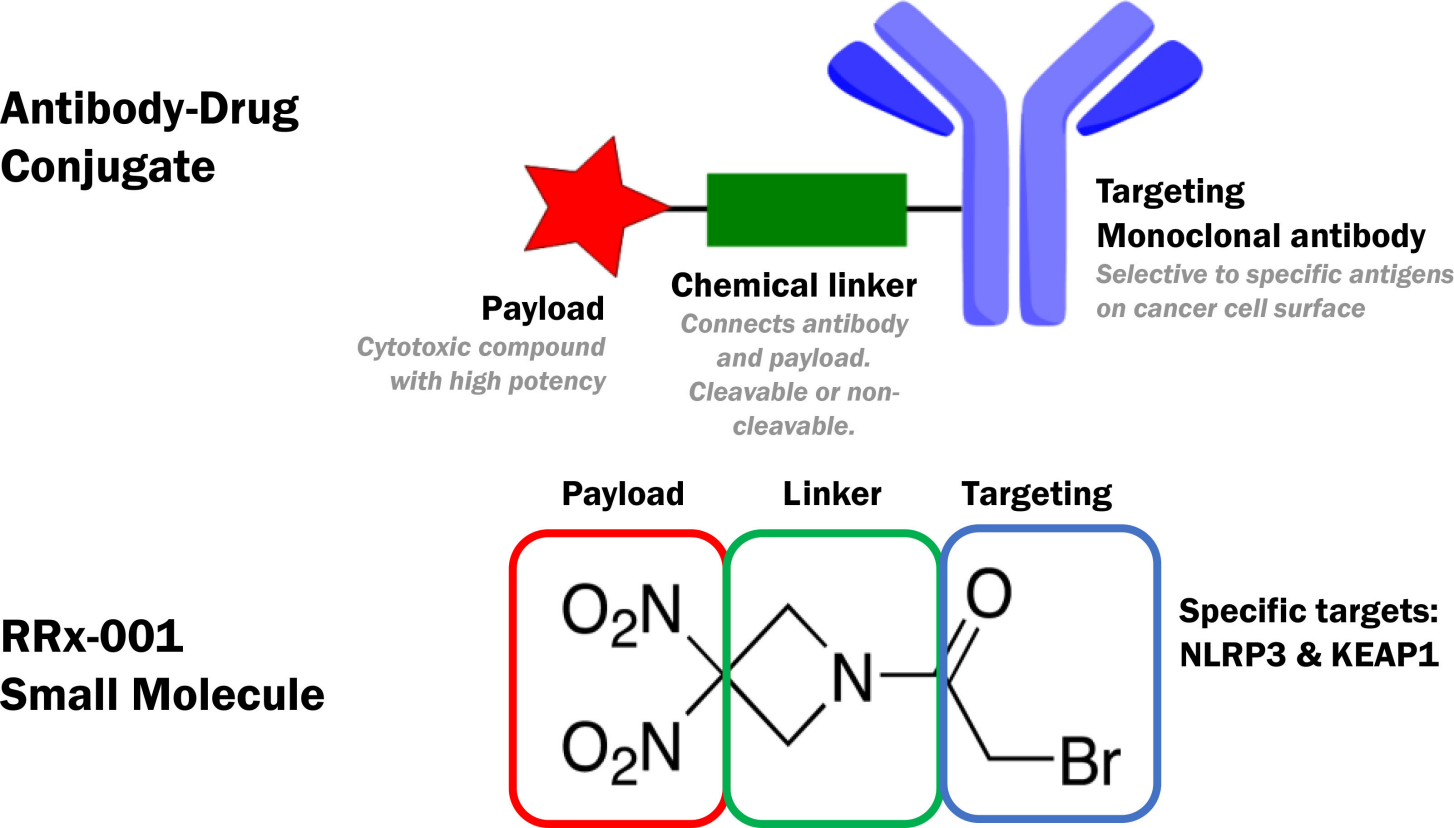
Engineering of RRx-001 to Resemble an Antibody Drug Conjugate (ADC).

RRx-001 amalgamates two pharmacophores with mixed and diametrically opposed biological functions: the targeting moiety is (mostly) anti-inflammatory/anti-oxidative and the payload moiety, depending on whether conditions are redox-reduced and hypoxic, is (mostly) pro-inflammatory/pro-oxidative. These two pharmacophores are an acyl bromide and a dinitroazetidine, which are conjugated through a stable i.e., non-cleavable amide linker. This design intentionally resembles that of an antibody drug conjugate (ADC), as shown below in **Figure 1**.

As structure dictates activity, the design of RRx-001 serves as a jumping off point to describe its paradoxical antioxidant/pro-oxidant and anti-inflammatory/proinflammatory properties *in vivo*.

Parenterally administered RRx-001 is currently in a Phase 3 trial with a platinum doublet (etoposide + carboplatin/cisplatin or EP) for the treatment of third line and beyond small cell lung cancer (SCLC) called REPLATINUM (3) and in a Phase 2b radioprotective trial called KEVLARx in newly diagnosed head and neck cancer. As an uncharged (albeit non-lipid soluble) small molecule, RRx-001 and its metabolites are cell membrane permeable (4) and cross the highly restrictive blood brain barrier (BBB) (5), which otherwise excludes 98% of small molecule drugs and ~100% of biologics (6, 7); this BBB permeability and encouraging evidence of clinical activity against brain metastases (5) and glioma (8, 9) prompted the preclinical assessment of RRx-001 in various neurodegenerative diseases, including Alzheimer’s, Parkinson’s, and ALS/MND where it has, to date, demonstrated genuine disease modifying potential (10).

It may seem counterintuitive that RRx-001, as a prooxidant, which reverses resistance to chemotherapy protects against the progression of neurodegenerative diseases in preclinical models since already elevated levels of inflammation and reactive oxygen and nitrogen species (RONS) are thought to underlie them. However, a common misconception is that RONS are universally harmful and that so-called “antioxidants” must scavenge them to be effective. In fact, like RRx-001, most antioxidants are either prooxidants that generate reactive species and/or electrophiles, which form covalent adducts with proteins. This is the case, for example, with physical exercise and several phytochemicals including curcumin from turmeric, diallyl sulfide from garlic, resveratrol from grapes, epicatechin from cocoa or green tea and sulforaphane from cruciferous vegetables like broccoli, cauliflower, and kale (11), all of which generate a mild oxidative stress that, in turn, upregulates endogenous antioxidant defense systems, such as reduced glutathione (12). This is also the case with dimethyl fumarate (DMF), an electrophilic compound, which has been approved for the treatment of relapsing multiple sclerosis in the United States and Europe, and which is under investigation in cancer and other neurodegenerative diseases like Alzheimer’s, and Parkinson’s (13). Like RRx-001, DMF induces an initial oxidative burst through alkylation of thiols and depletion of glutathione (GSH) that activates Nrf2 and ultimately increases GSH levels (14).

Structure activity relationships have demonstrated that analogs of RRx-001, which do not contain the inflammatory payload are minimally active.

**Figure 2:**
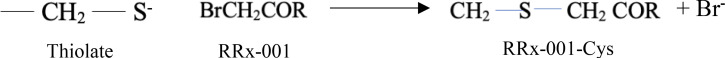
RRx-001 Reacts Rapidly with Thiolate Anions.

The α-bromoacetamide moiety of RRx-001 selectively and rapidly alkylates/derivatizes available thiolate anions (R-S^-^) through nucleophilic substitution (S_N_2) which displaces the leaving group, bromide (15), as shown in **Figure 2**. A thiolate is much more nucleophilic and reactive than a neutral thiol (16). The stability of the thiolate residues to which RRx-001 preferentially binds depends on the presence and proximity of cationic amino acids or specific hydrogen bonds that depress their pK_a_ value and, hence, increase reactivity (4).

Depending on the cysteine thiolate residues that it alkylates, RRx-001 irreversibly inhibits the function of proteins. Because RRx-001 binds covalently, its half-life is effectively equivalent to the resynthesis half-life of the bound protein, which impacts the dosing frequency. Accordingly, RRx-001 is administered on a weekly or monthly basis, depending on the disease indication. The fact that RRx-001 only reacts with a few select thiolates (17) based on metabolism and disposition studies (18) probably accounts for the absence of dose limiting toxicities, and drug-related serious adverse events (SAEs) in over 350 patients treated with it to date. Also, a maximal tolerated dose (MTD) has never been reached (19). The main adverse event associated with RRx-001 is an infusion-related superficial thrombophlebitis-like venous inflammation and pain (20), which is treated, if it is treated at all, with non-steroidal anti-inflammatory drugs. RRx-001 is administered in an ex vivo device with an aliquot of anticoagulated blood to improve patient discomfort and to prevent more serious complications and sequelae such as infection or progression to deep vein thrombosis. However, typical chemotherapy-like hematologic and non-hematologic adverse events such as nausea/vomiting, alopecia, weight loss, fatigue, stomatitis, diarrhea, and myelosuppression are never encountered with it. In addition to venous inflammation, another common side effect with RRx-001 is tumor pseudoprogression, in which decreased tumor burden follows transient tumor growth due to edema and immune infiltration (21). Pseudoprogression mimics true early progression, which is potentially problematic because it may lead to premature discontinuation from treatment with RRx-001 (22).

One of the most important targets of RRx-001 is the nucleotide-binding oligomerization domain, leucine-rich repeat, and pyrin domain containing 3 (NLRP3) inflammasome. RRx-001 is a double inhibitor of the NLRP3 inflammasome, firstly because it selectively binds to cysteine 409 on the central NACHT domain of NLRP3 (14, 15), which prevents its assembly and, secondly, because it inhibits nuclear factor kappa B (NF-κB) (23).

## 2 Targeting moiety

### 2.1 Double inhibition of the NLRP3 inflammasome by RRx-001

The NLRP3 inflammasome is an intracellular multiprotein complex that activates in response to harmful stimuli, such as dead cells, irritants, or pathogens. This activation mediates an inflammatory response through the production and release of IL-1β, IL-18, and gasdermin D (GSDMD).

Inflammation is the first line of defense against infection, which makes NLRP3 inflammasome activation beneficial and homeostatic, provided that the duration of the subsequent inflammatory response is short-lived i.e., days to weeks, and resolution quickly follows removal of the noxious stimulus (24). However, if the inflammatory response is inadequate or if the noxious stimulus persists, chronicity develops, which is pathologic and maladaptive, because of the overproduction of reactive oxygen species (ROS) and cytokines from ongoing inflammasome activation and immune cell infiltration (25). A malicious cycle of chronic inflammation, oxidative stress, and destruction of healthy cells and tissues ensues, which over time i.e., months to years leads to disease, end-organ damage, and even mortality.

**Figure 3:**
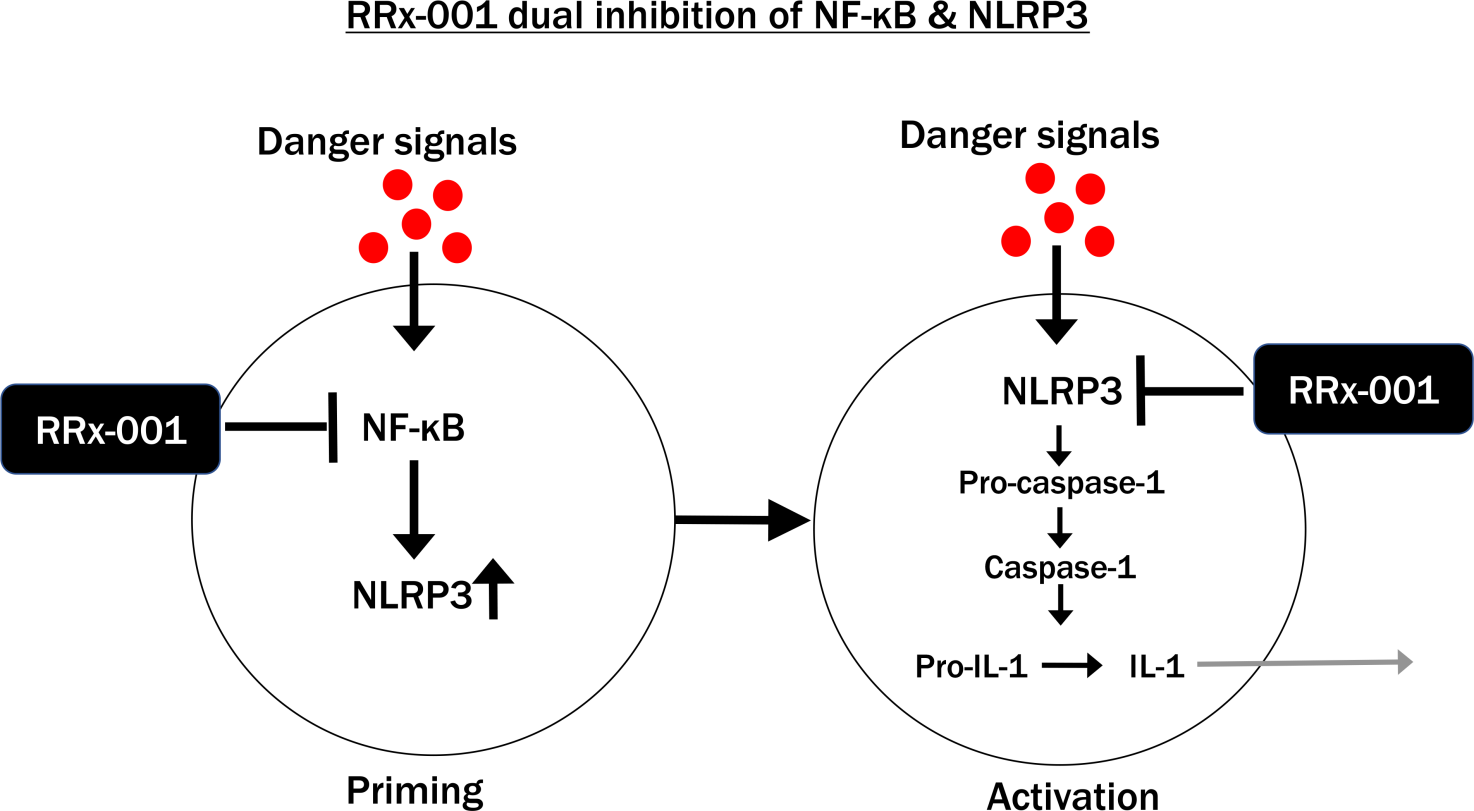
RRx-001 Inhibits Both Steps of NLRP3 Inflammasome Activation.

NLRP3 inflammasome activation is canonically a two-step process involving NF-κB priming from a range of pathogen-associated molecular patterns (PAMPs) such as viral and bacterial components and damage-associated molecular patterns (DAMPs) (Step 1) and protein complex assembly (Step 2), also involving a range of PAMPs and DAMPs, which are also known as “danger signals” (26). As shown in **Figure 3**, RRx-001 inhibits both steps, due to its repression of TAK1, which activates NF-κB, and NLRP3 assembly. The non-canonical NLRP3 pathway involves human caspase-4 and caspase-5 from gram-negative bacterial infection (27).

Aberrant activation of the NLRP3 inflammasome is associated with the onset and progression of many diseases including metabolic syndrome, type 2 diabetes, non-alcoholic fatty liver disease, cardiovascular disease, chronic kidney disease, cancer, depression, neurodegenerative and autoimmune diseases, and endometriosis. For this reason, NLRP3 inflammasome inhibitors are used to treat a range of diseases from cancer to neurodegenerative conditions like Parkinson’s and Alzheimer’s (28).

Indeed, outside of cancer, RRx-001, the most clinically advanced of the direct NLRP3 inhibitors, including MCC950, which is no longer in development, has received FDA Fast Track status (29) as a radioprotector to prevent/ameliorate severe oral mucositis based on the results of a randomized Phase 2 clinical trial called PREVLAR (30) and is also under preclinical study in Parkinson’s Disease and amyotrophic lateral sclerosis (ALS)/motor neuron disease with grants awarded from the Michael J. Fox Foundation (MJFF) and Fight MND, respectively. The second most clinically advanced NLRP3 inflammasome inhibitor is OLT-1177 (Dapsutrile), having completed at least three Phase 2 trials in gout, osteoarthritis, and heart failure (31). On top of NF-κB and NLRP inhibition, RRx-001 directly and indirectly upregulates the master antioxidant transcription factor, nuclear factor (erythroid-derived 2)-like 2 (Nrf2), which differentiates RRx-001 from other rationally designed, unimechanistic direct NLRP3 inhibitors like selnoflast, dapsutrile, DFV890 (IFM-2427), and ZYIL1 (32). Besides NLRP3 inhibition and Nrf2 induction, other established mechanisms of action for RRx-001 including epigenetic modulation (33), tumor associated macrophage repolarization (34), and vascular normalization (6), which may play greater or lesser roles depending on the disease indication, are not discussed herein since this review was solicited specifically for a redox-based article collection.

### 2.2 Double upregulation of Nrf2 by RRx-001

Because RRx-001 reacts with glutathione and cysteine (preferentially in their thiolate forms), it induces oxidative stress (OS) (30). In response to OS, which damages cellular components such as proteins, DNA and lipids the constitutive inhibitor of Nrf2, Kelch-like ECH-associated protein 1 (KEAP1), physically dissociates from Nrf2, leading to the nuclear translocation of Nrf2, and transcription of a detoxifying battery of antioxidant response element (ARE) genes such as reduced glutathione (GSH), glutathione S-transferase Ya, NAD(P)H oxidoreductase (NQO1) and heme oxygenase-1 (HO-1) (35). In addition to oxidation of specific cysteine residues in KEAP1, RRx-001 is thought to form adducts with these residues, presumably leading to the ubiquitination and subsequent proteasome-dependent degradation of KEAP1.

The preclinical and clinical radio- and chemoprotective effects of RRx-001 that have been observed both preclinically and clinically are attributable not only to NLRP3 inflammasome inhibition but also to Nrf2 induction (18, 36, 37). RRx-001 is such a strong activator of Nrf2 that it significantly induces the transcription of target genes including heme oxygenase-1 (HO-1) even in the presence of N-acetyl-l-cysteine or glutathione (GSH) (37–39). This hyperactivation of Nrf2 raises the question whether RRx-001 administration has the potential to protect tumors both from chemotherapeutic agents and radiation-induced damage since Nrf2 is possibly protumorigenic. Preclinical data have established that, in fact, RRx-001 selectively kills Caco-2, A2780 (ovarian cancer), and UWB1 (BRCA1-null human ovarian cancer) cells but not CRL-1459/CCD-18Co normal fibroblast colon cells (35) and clinical data support the anticancer activity of RRx-001 both alone (19) and in combination with chemotherapy (40), immunotherapy (41), and radiation (42). Separate from all that, however, it is persistent, long-term activation of Nrf2 that seems to protect cancer cells from genotoxic chemo- and radiotherapies, and to make them refractory to treatment, not controlled, intermittent activation from a small molecule Nrf2 inducer like RRx-001 (43). Also, Nrf2 is thought to suppress carcinogenesis especially at early stages, owing to its detoxifying activity (44).

RRx-001 is only behind dimethyl fumarate (DMF, BG-12, Tecfidera^®^) and omaveloxolone (Skyclaris^®^), which are approved for the treatment of remitting-relapsing multiple sclerosis (RRMS) and psoriasis (DMF), and Friedrich’s ataxia (omaveloxolone) in terms of its clinical advancement (45). Like RRx-001, omaveloxolone is well tolerated and so is DMF except for one rare serious adverse event, progressive multifocal leukoencephalopathy (PML), an opportunistic and often fatal infection of the brain (46).

As previously stated, because of its reactivity with the thiolate forms of cysteine and glutathione, RRx-001 significantly induces oxidative stress (OS), at least initially, which is a double-edged sword, because RRx-001 may exacerbate already high baseline OS levels in older individuals and/or those with chronic diseases like cancer, heart failure, and diabetic nephropathy before the compensatory expression of Nrf2-related cytoprotective enzymes such as catalase, glutathione S-transferases, glutathione reductases, glutathione peroxidase-1 (GPx), heme oxygenase-1, superoxide dismutase (SOD), thioredoxin, and quinone oxidoreductases occurs (47).

## 2 Non-cleavable linker

In antibody drug conjugates (ADCs) a linker connects the targeting moiety with the payload. Linkers are classified as cleavable and non-cleavable. The amide linker in RRx-001 is stable to external enzymatic cleavage. The comparative advantage of a non-cleavable linker over a cleavable one in this case is increased plasma stability so that the dinitroazetidine payload is not prematurely released or released in normal cells.

## 3 Payload moiety

It is perhaps misleading to only refer to the highly strained dinitroazetidine end of RRx-001 as the “payload” given the enhanced biologic activity of the “targeting moiety” against NF-κB/NLRP3 inflammasome and KEAP1, the negative regulator of Nrf2. Nevertheless, the strain in the 4-membered dinitroazetidine and its reactivity with GSH makes it susceptible to nucleophilic ring opening and strain release transformations *in vivo* under ischemic/hypoxic conditions. Cleavage of C-C and C-NO_2_ bonds has the potential to generate carbon-centered radicals and nitric oxide (NO) or NO-related species, such as the nitrosonium ion (NO^+^) or the nitroxyl anion (NO^-^), respectively, which underlie the anticancer DNA damaging effects of RRx-001. X-ray crystal structure analysis on RRx-001 demonstrates that the azetidine ring is puckered to reduce steric and electronic repulsions, which favors ring opening to relieve strain (48).

Preclinical and clinical data have demonstrated that RRx-001 is an ‘on-demand’ nitric oxide (NO) donor and superagonist, meaning that, unlike the organic nitrates, which are commonly used in the treatment of cardiovascular disease, and other nitric oxide donors such as furoxans, benzofuroxans, NONOates, and S-nitrosothiols, NO is released from RRx-001 at high levels not systemically, but locally and only where ischemia/hypoxia is present, that is in the right time and right place (49, 50). This local release obviates the toxicities such as hypotension, methemoglobinemia, dizziness, nausea, and headache that are associated with these other nitric oxide donors. In solid tumors, where hypoxia is common, RRx-001-mediated NO donation has been shown preclinically and clinically to dilate the vasculature (51) and to augment the delivery of oxygen (52), other anticancer drugs (6), and effector cells as a result (53). Also, in tumors with high levels of oxidative stress, nitric oxide, a highly diffusible and reactive free radical, combines with superoxide to form the powerful oxidant, peroxynitrite (ONOO^-^) (47), which induces DNA damage; peroxynitrite is also associated with macrophage cytotoxicity since immune myeloid cells, like macrophages, produce both nitric oxide and superoxide to generate it (54). Preclinical data have demonstrated that the application of hyperthermia also increases NO production from RRx-001.

In addition, RRx-001 derivatizes deoxyhemoglobin and displaces nitric oxide from its binding site on beta cysteine 93; this adds to the local overproduction of NO since these RRx-001-derivatized red cells preferentially adhere to hypoxic/ischemic vasculature (55–57).

In addition to cancer (58), nitric oxide insufficiency/deficiency is a characteristic of several disease states (59) including pulmonary hypertension, hyperlipidemia, and non-alcoholic steatohepatitis (NASH) (60), COVID-19 (61, 62), chronic kidney disease, myocardial infarction (63), cerebral malaria and stroke (64), ischemia reperfusion injury (65), hemorrhagic shock (66), and sickle cell disease (67) where RRx-001 has demonstrated activity, although not all this data is publicly available.

However, the effects of nitric oxide are not one-sided in terms of its pro-oxidant properties, as NO also induces transcriptional upregulation of Nrf2-related protective genes and activation of the tumor suppressor, p53 (68, 69).

## 4 Conclusion and future directions

The paradox of RRx-001, and what separates it from other NLRP3 inflammasome inhibitors and Nrf2 inducers, several of which are rationally designed to only inhibit the NLRP3 inflammasome or KEAP1, for example, is that RRx-001 switches between pro-oxidant/proinflammatory activity and antioxidant/anti-inflammatory activity depending on the redox potential of the cellular environment and the presence or absence of hypoxia. Such plasticity is rarely, if ever, seen because small molecules do not tend to alter their mechanism of action from one tissue to another. Thus, as a rule, a protective drug universally protects, no matter the tissue type, and the same is true for a cytotoxic one, which damages diseased and healthy cells alike. Chemotherapy may appear to preferentially target cancer cells but that is only because rapidly dividing cells are more sensitive to its toxic effects. In fact, chemotherapy is non-specific; it acts on rapidly dividing cancer cells and rapidly dividing normal ones, like those in the hair follicles, the gastrointestinal tract, and the bone marrow; hence the common side effects from chemotherapy of hair loss, vomiting and/or diarrhea, and myelosuppression.

**Figure 4:**
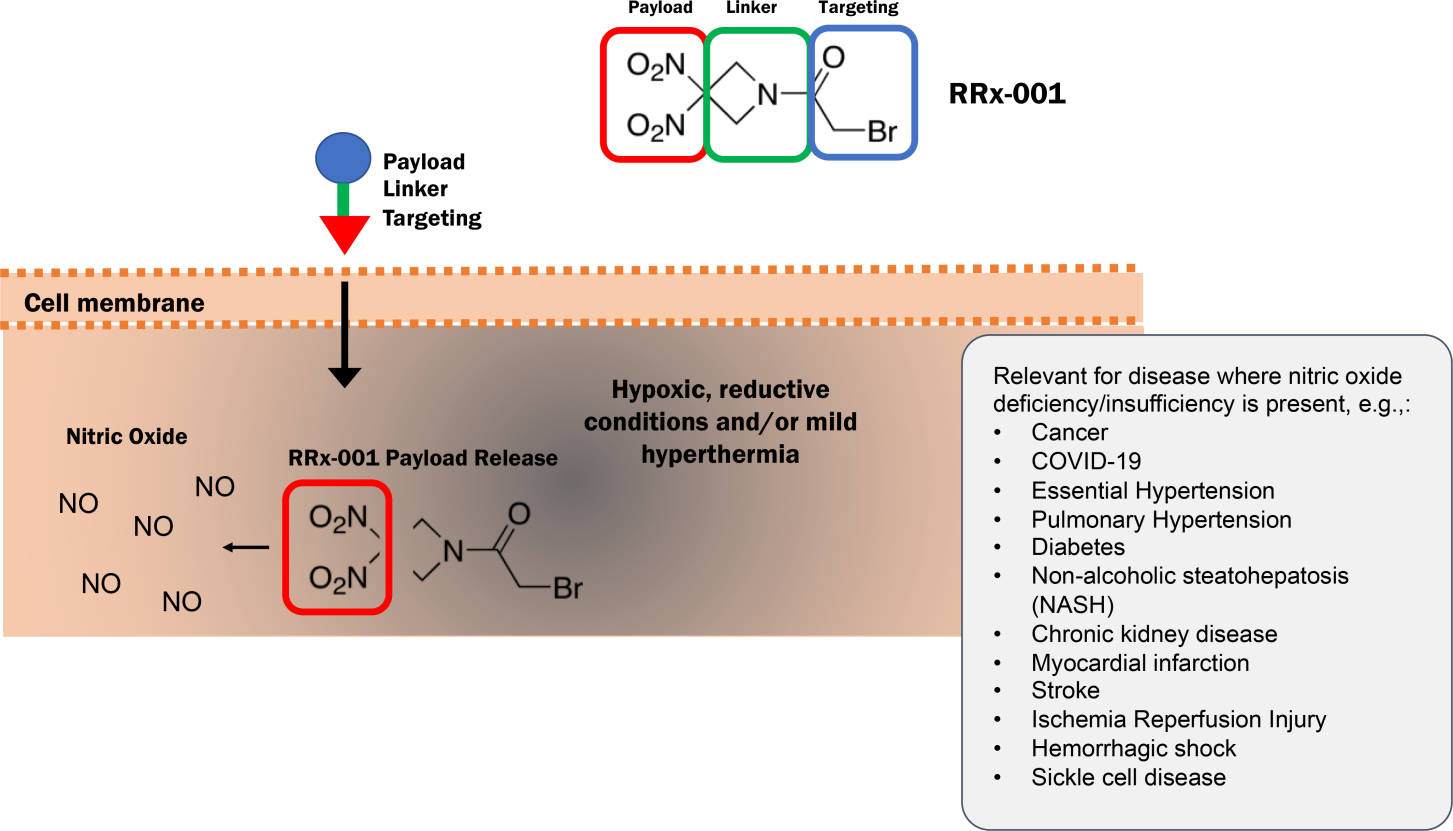
Dinitroazetidine Ring Fragmentation and Release of Nitric Oxide Under Reductive Conditions in Hypoxic Tissues.

The plasticity of RRx-001 is design-driven: the dinitroazetidine ring is stable under normoxia but under hypoxic, reductive conditions where vasodilation is needed most to increase local blood flow and oxygenation the ring fragments and releases nitric oxide *via* a radical process. Under mild hyperthermia, RRx-001 also increases NO production (70). See **Figure 4** below. In hypoxic tumors where high levels of superoxide anion ${\text{O}}_{2}^{-}$ are an observed hallmark, NO outcompetes the enzyme superoxide dismutase (SOD), which breaks down superoxide into oxygen and hydrogen peroxide, and readily combines with ${\text{O}}_{2}^{-}$ to produce the cytotoxic and genotoxic radical, peroxynitrite, as follows: ${\text{O}}_{2}^{-}+\text{NO}\to {\text{ONOO}}^{-}$ (71, 72). In turn, peroxynitrite decomposes to nitrogen dioxide (NO_2_) and hydroxyl radicals (·OH), two very potent oxidants with significant cytotoxic potential.

Even though RRx-001 mediates nitro-oxidative stress and is cytotoxic to tumors through the formation of ONOO^−^ and carbon- and nitrogen-centered radicals, it also protects against ischemia reperfusion injury (IRI) in several organs, including the heart and the kidneys, as preclinical data have demonstrated (73). One of the likely mechanisms behind this protective effect is NO release from RRx-001, which is thought to suppress superoxide generation through S-nitrosylation (SNO) of mitochondrial complex I. As a gas, nitric oxide, which passively diffuses through mitochondrial membranes, reversibly S-nitrosates cysteine 39 on the ND3 subunit of mitochondrial complex I (NADH:ubiquinone oxidoreductase); complex I is a dynamic enzyme, which transitions between active (-A) and deactivated (-D) states. During ischemia, complex I deactivates (-D state), which exposes the ND3 subunit Cys39 residue and makes it susceptible to modification. During reperfusion, complex I reactivates (-A state), which leads to an oxidative burst. S-nitrosylation of ND3 cysteine 39 temporarily inhibits complex I activity and delays mitochondrial recovery at the onset of reperfusion, hence attenuating excessive reactive oxygen species (ROS) production and oxidative damage (74).

The bromoacetyl end of the RRx-001 molecule inhibits the NLRP3 inflammasome, which is responsible for the release of proinflammatory cytokines, and KEAP1, which sequesters the antioxidant powerhouse, Nrf2, and targets it for degradation. NLRP3 inflammasome inhibition and Nrf2 induction are also related to the protective effects of RRx-001.

RRx-001 arose from a collaboration between the biopharmaceutical company, EpicentRx, (formerly RadioRx) and chemists from the aerospace and defense industry; the intent of this collaboration was to translate the well-known phrase, “the whole is greater than the sum of its parts” through a chimeric ADC-like small molecule, which combined independent chemical pharmacophores that were (and are) used in the aerospace and defense industry. Structure activity relationship (SAR) studies demonstrate that this is the case since the activity of the parent molecule significantly exceeds that of its individual pharmacophores or close analogs in which these pharmacophores are replaced (4).

Accordingly, RRx-001 is an entirely new molecular entity (NME) without precedent in the pharmaceutical space, which warrants the new USAN name, bromonitrozidine, reflective of its first-in-class mechanisms of action and its lack of belonging to any other pharmacological groups; the closest relative of RRx-001 is the high density, melt-castable explosive 1,3,3-Trinitroazetidine (TNAZ), which has been proposed as a replacement for dynamite (75). Like TNAZ, RRx-001 is extremely energetic, which makes it hazardous to manufacture, requiring special safety measures (76). These measures include the use of proper facilities in isolated areas well away from any habitation, and the need for substantial personal protective equipment and emergency procedures on site. Fortunately, the addition of solvents such as DMSO or polyethylene glycol (PEG) desensitizes RRx-001 and renders it safe for transport and use.

In summary, then, RRx-001 is a chimeric, CNS-penetrant (77), thiolate-reactive molecule that undergoes a physical change in response to an intrinsic, chemical trigger, reductive hypoxia (78). A potential second trigger is the application of mild hyperthermia. RRx-001 is active in cancer with a half maximal inhibitory concentration (IC_50_) in the subnanomolar range and synergizes with (79) and resensitizes to chemotherapies, immunotherapies, targeted therapies, and radiation (33, 80, 81). It is also a potential medical countermeasure against the effects of high dose, whole-body radiation exposure in radiological or nuclear incidents and has demonstrated preclinical neuroprotective effects in Parkinson’s and Alzheimer’s Diseases, Multiple Sclerosis, and Amyotrophic Lateral Sclerosis/Motor Neuron Disease.

In addition to RRx-001, co-crystals of RRx-001 and other dinitroazetidine-based small molecules with multi-indication potential are under development as is an oral formulation since in some disease indications, not necessarily cancer, the p.o. route is a more acceptable and economical method of administration despite the potential loss of bioavailability. A future patent-protected strategy (81) to increase the nitric oxide generation of RRx-001 (82–84), which takes advantage of its thermal sensitivity, is to apply mild hyperthermia noninvasively to the organs or tissues of interest during RRx-001 administration.

## Author contributions

Conceptualization, BO and TR. Methodology, BO and TR. Writing—original draft preparation, BO and TR. Review and editing, all authors. All authors contributed to the article and approved the submitted version.
